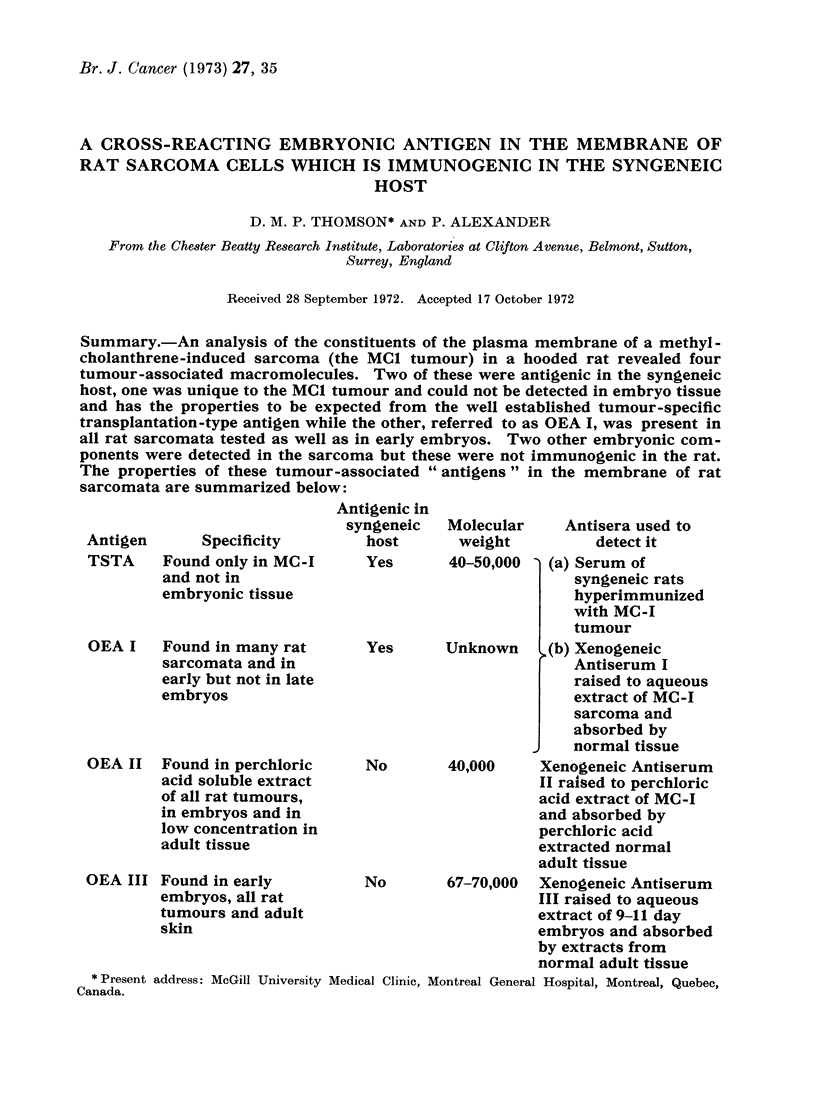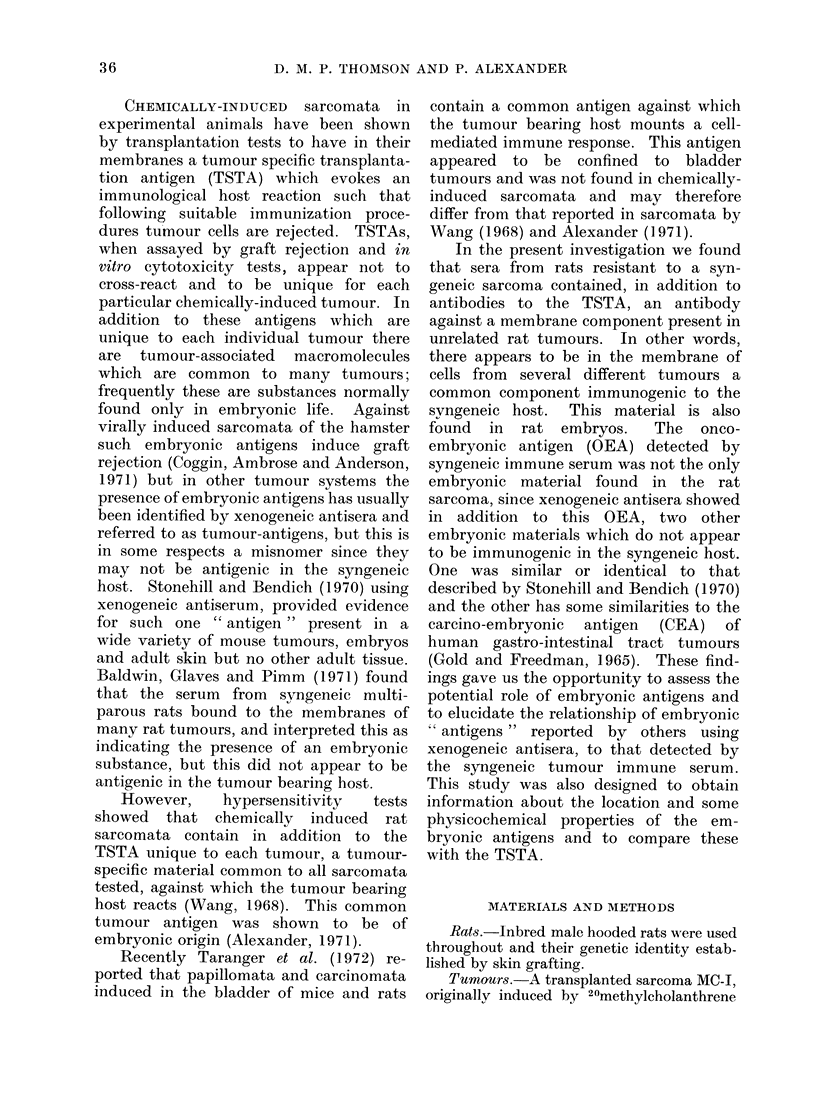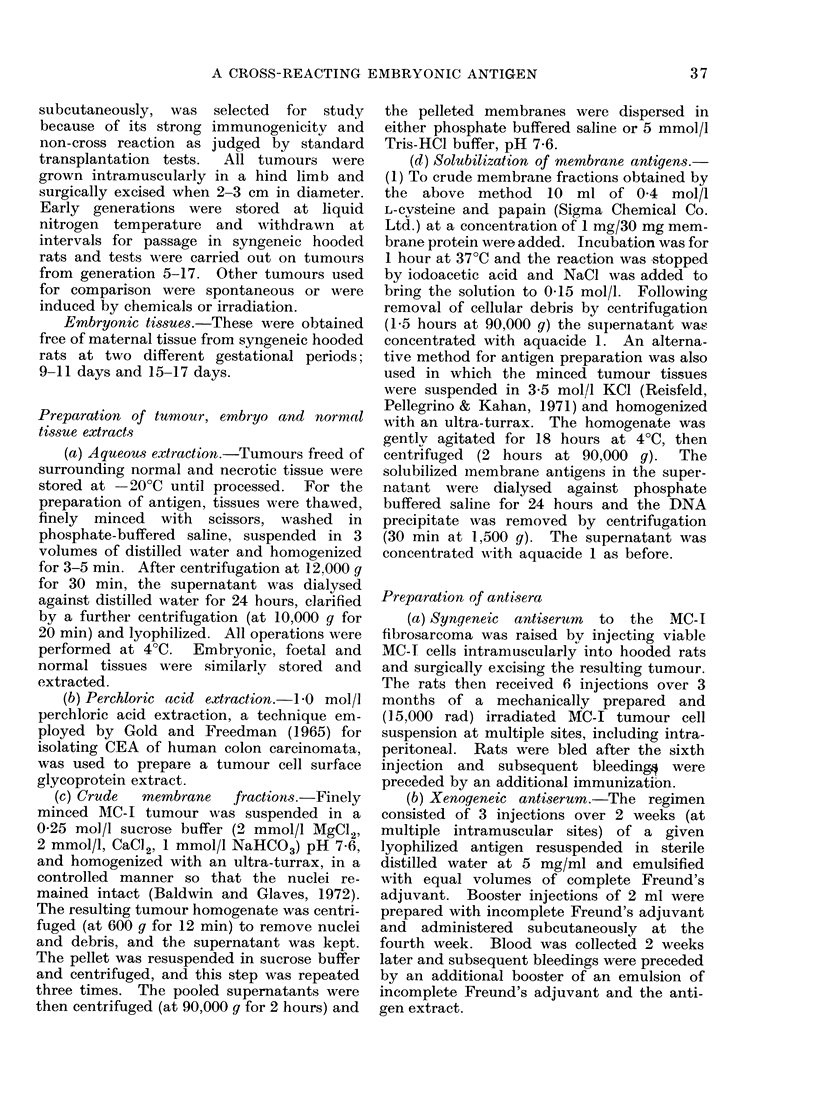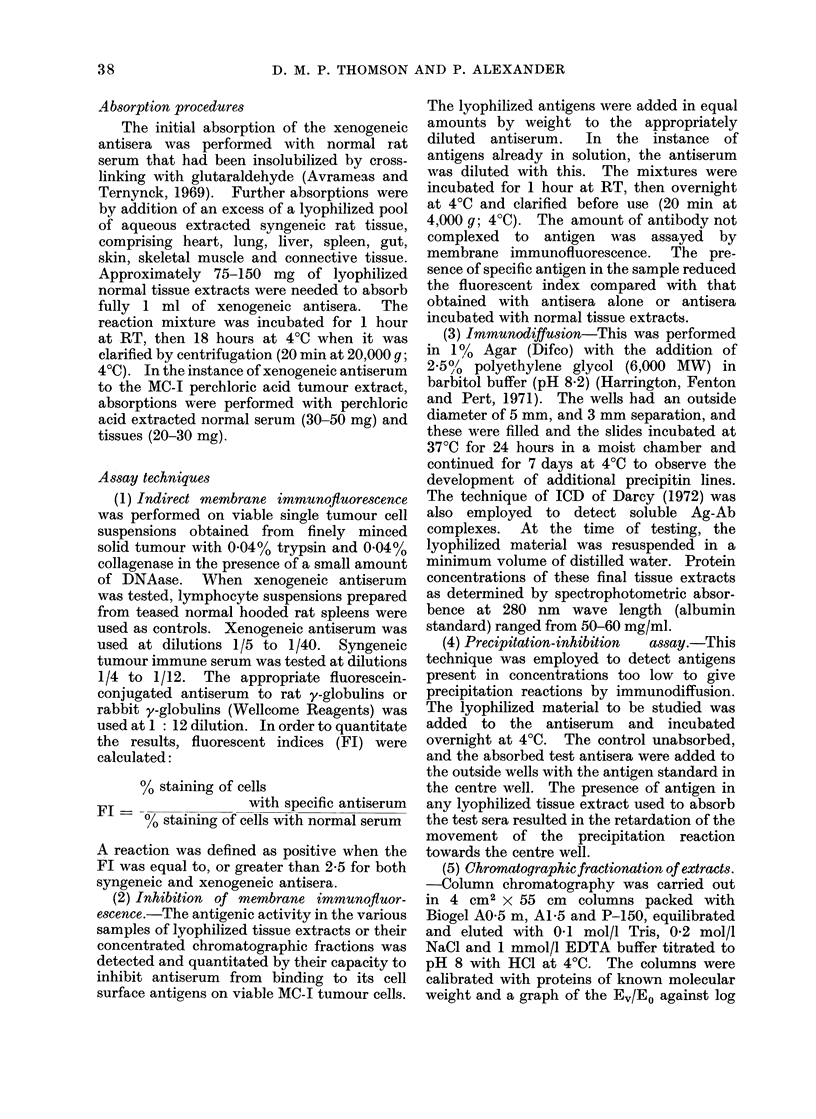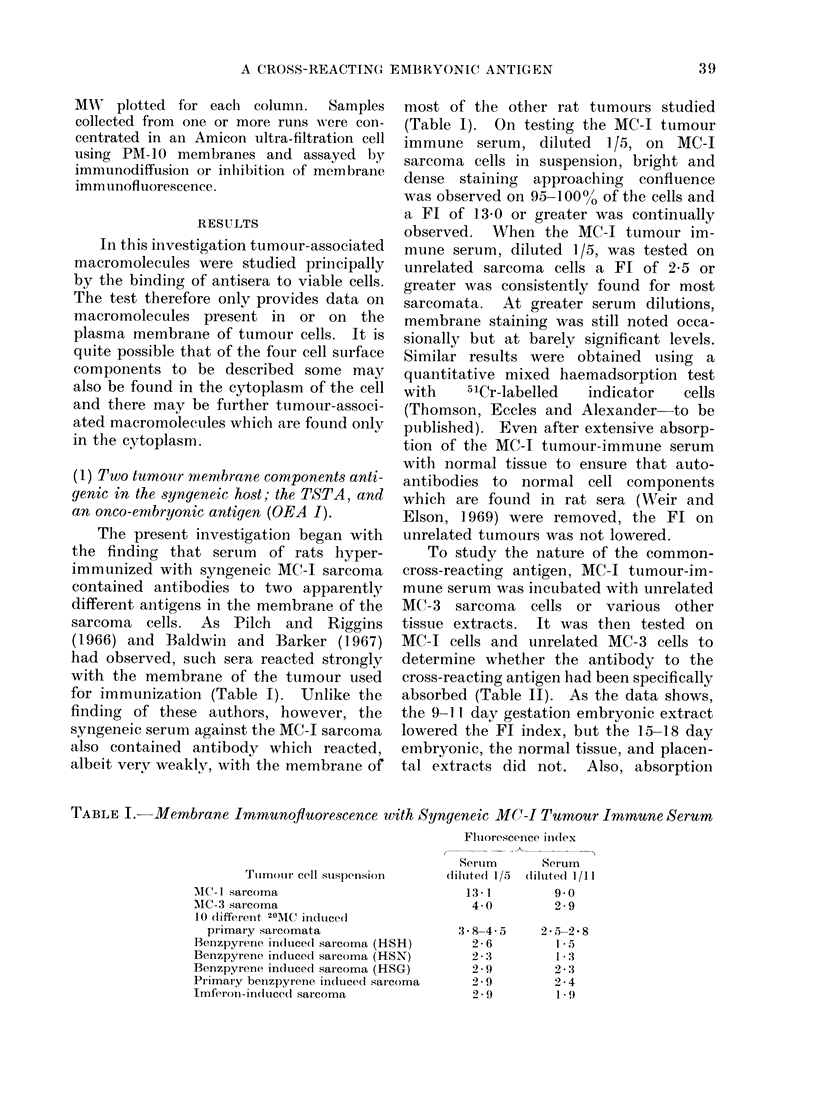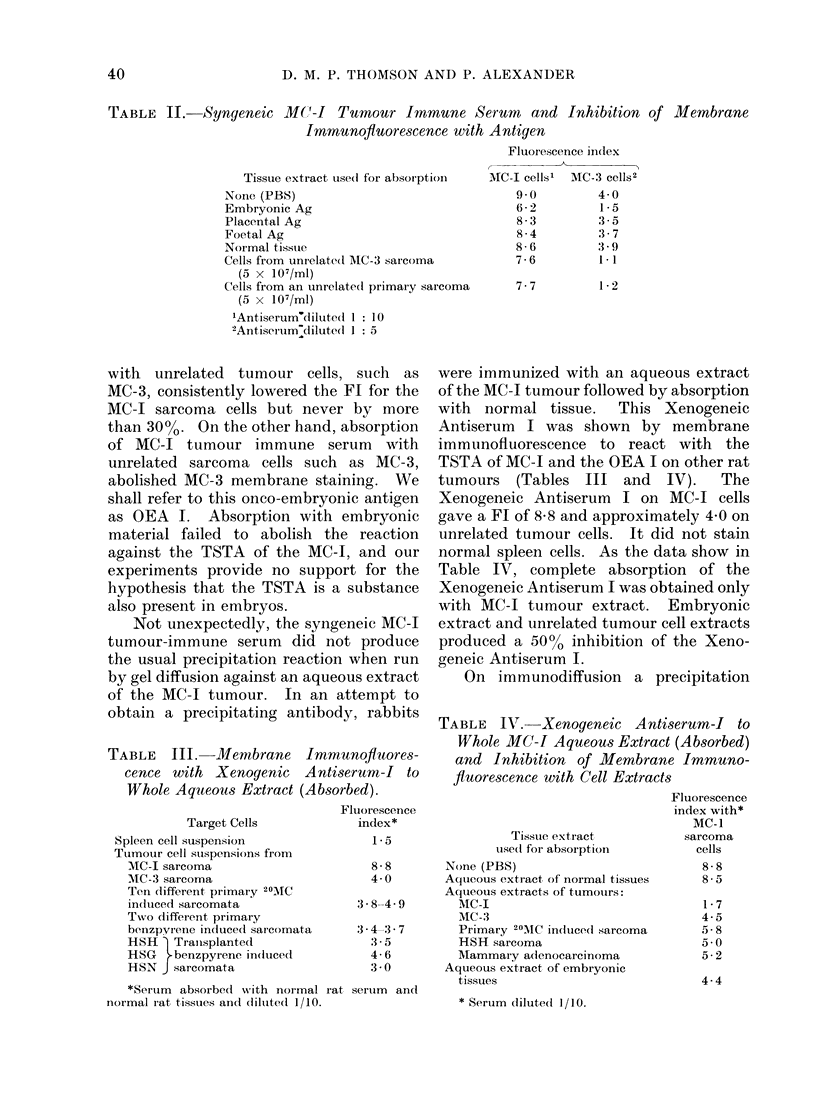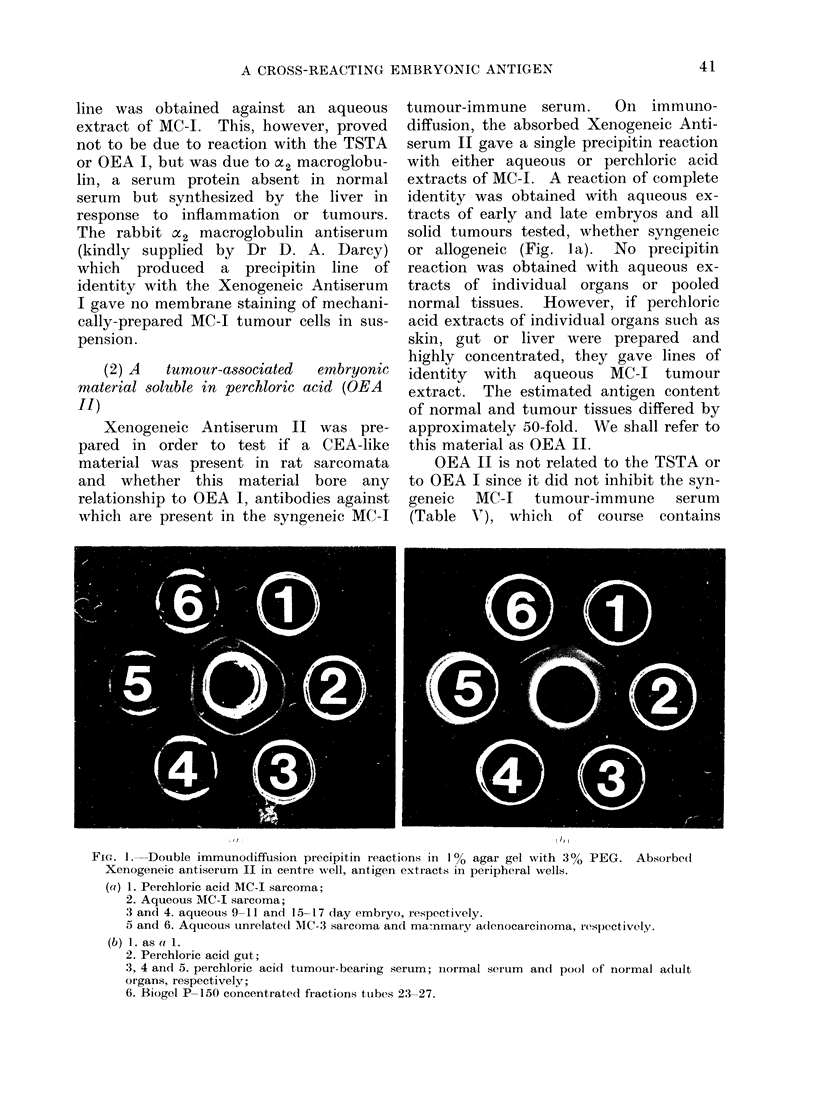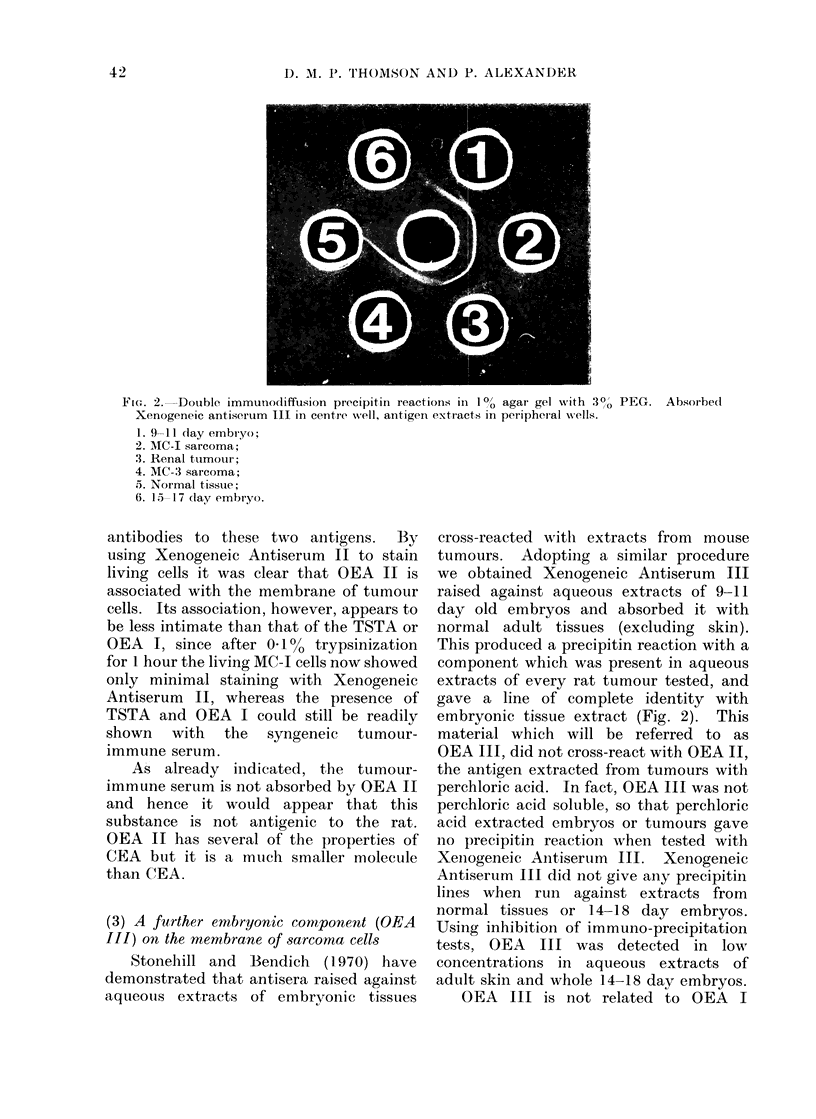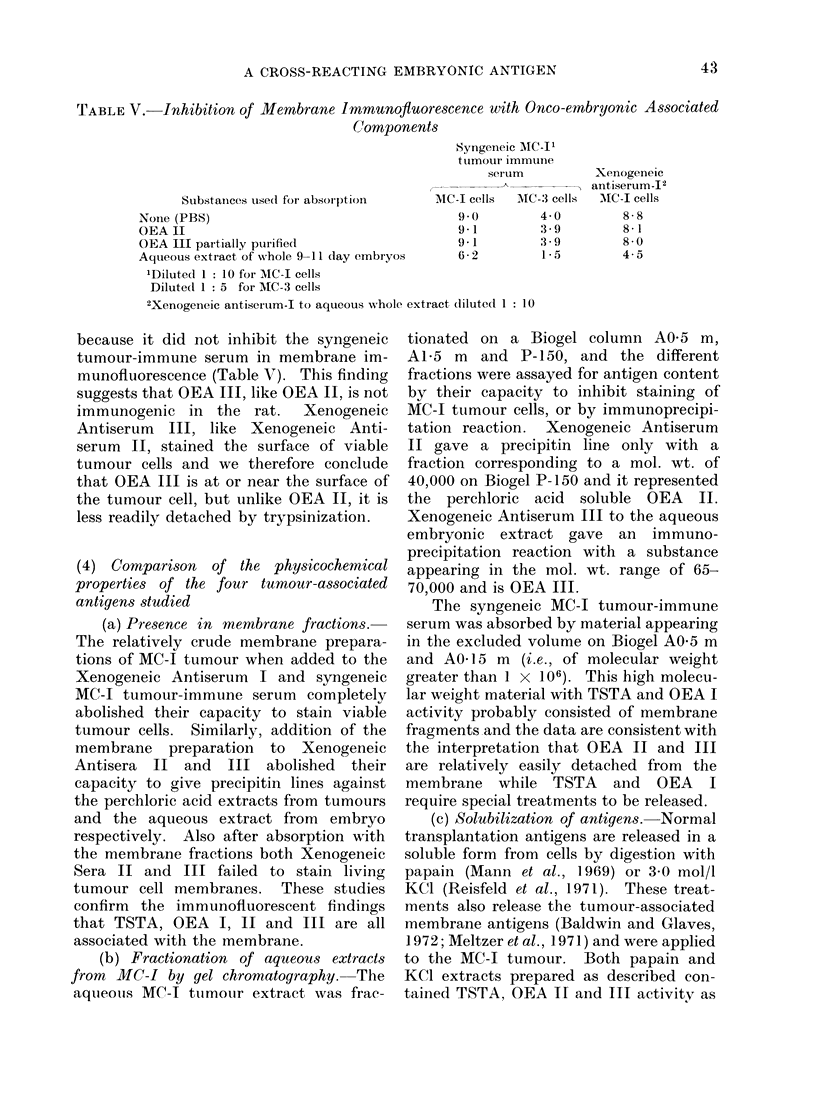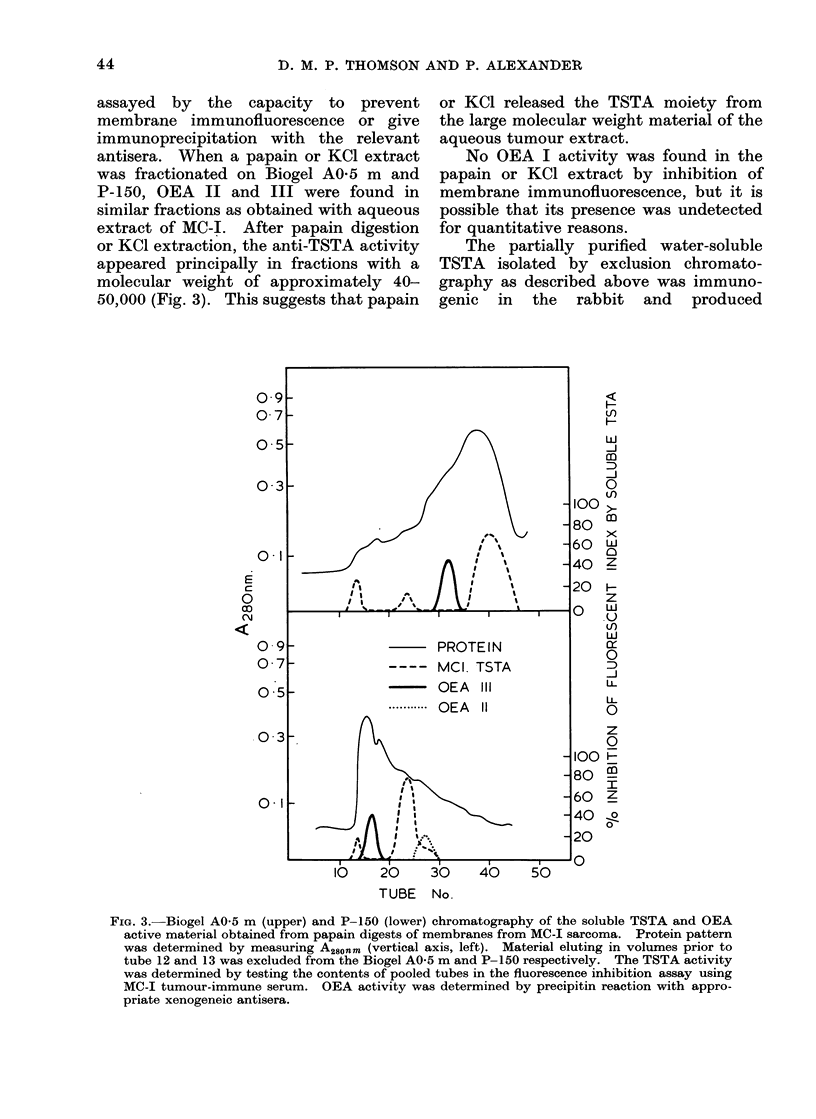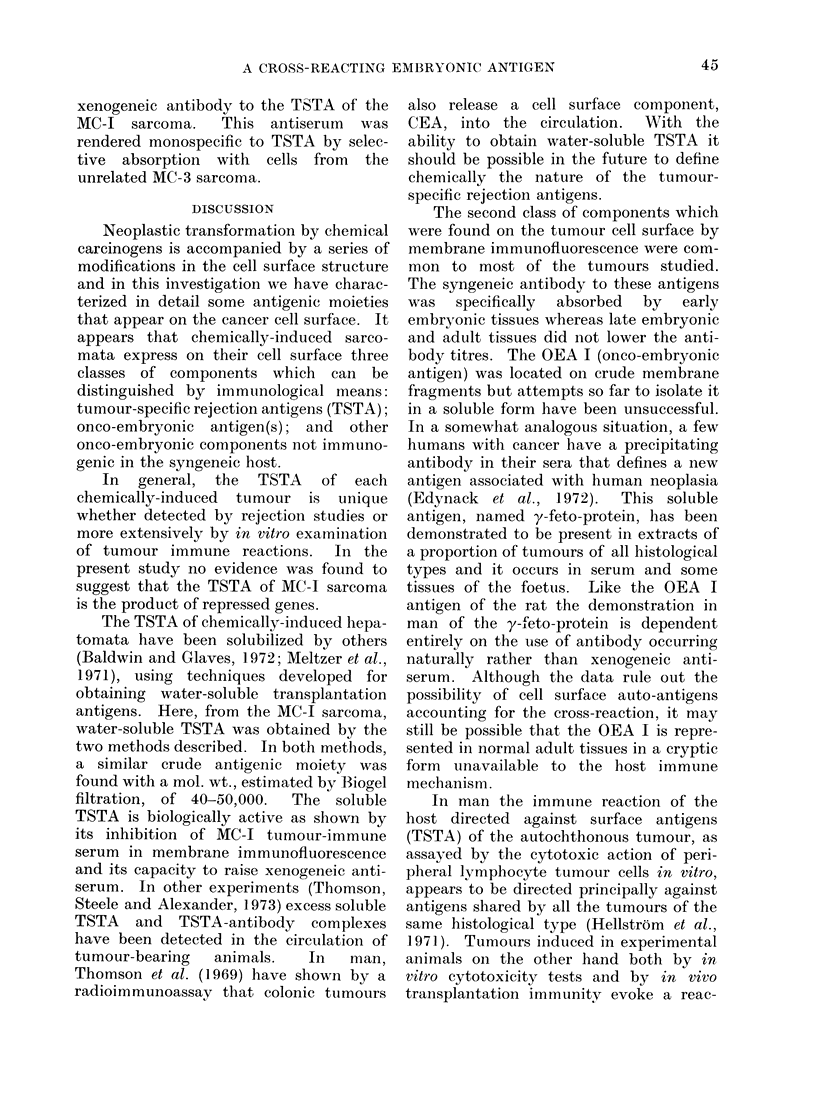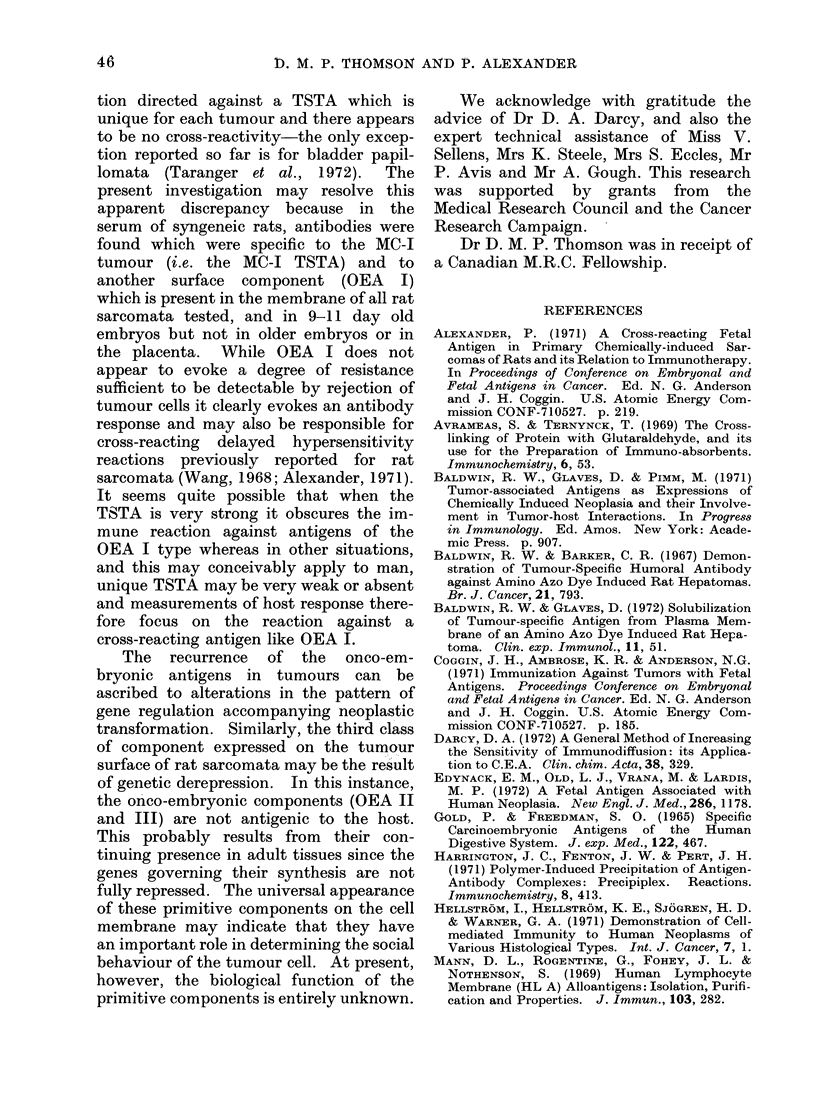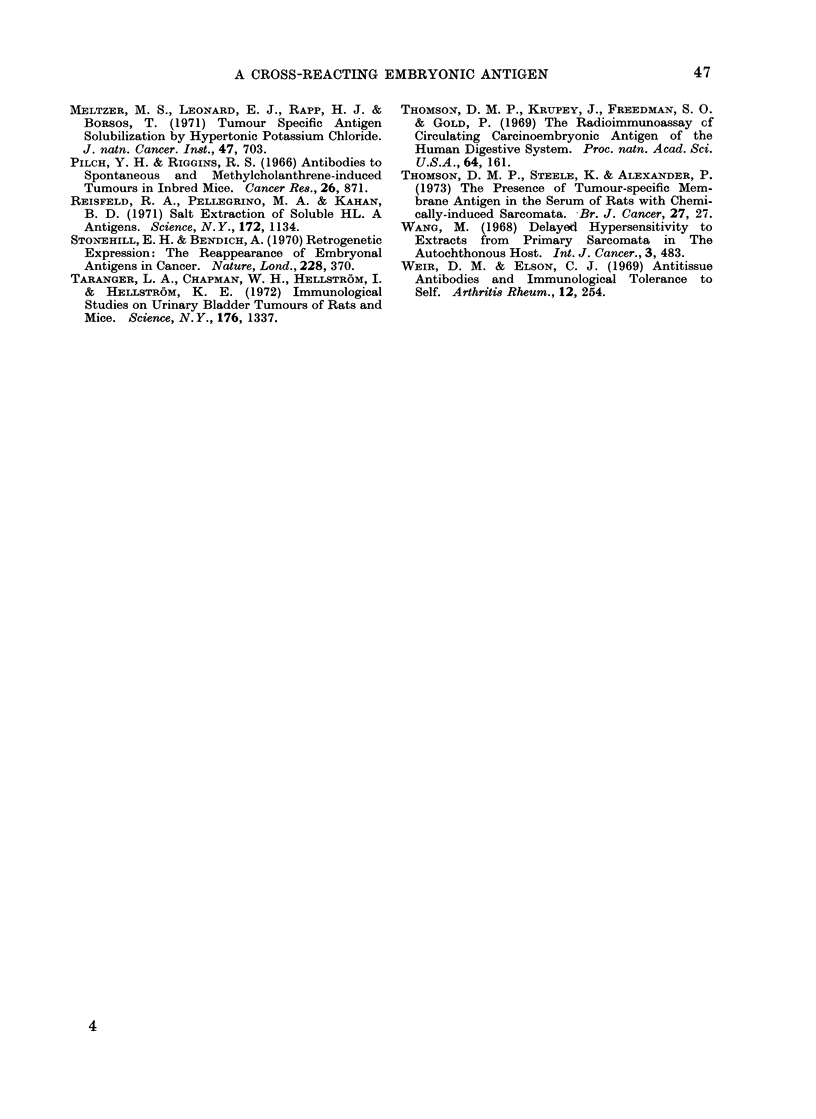# A Cross-Reacting Embryonic Antigen in the Membrane of Rat Sarcoma Cells which is Immunogenic in the Syngeneic Host

**DOI:** 10.1038/bjc.1973.5

**Published:** 1973-01

**Authors:** D. M. P. Thomson, P. Alexander

## Abstract

**Images:**


*Br. J. Cancer* (1973) **27**, 35

### A CROSS-REACTING EMBRYONIC ANTIGEN IN THE MEMBRANE OF RAT SARCOMA CELLS WHICH IS IMMUNOGENIC IN THE SYNGENEIC HOST

D. M. P. THOMSON* AND P. ALEXANDER

*From the Chester Beatty Research Institute, Laboratories at Clifton Avenue, Belmont, Sutton, Surrey, England*

Received 28 September 1972. Accepted 17 October 1972

**Summary.—An analysis of the constituents of the plasma membrane of a methylcholanthrene-induced sarcoma (the MC1 tumour) in a hooded rat revealed four tumour-associated macromolecules. Two of these were antigenic in the syngeneic host, one was unique to the MC1 tumour and could not be detected in embryo tissue and has the properties to be expected from the well established tumour-specific transplantation-type antigen while the other, referred to as OEA I, was present in all rat sarcomata tested as well as in early embryos. Two other embryonic components were detected in the sarcoma but these were not immunogenic in the rat. The properties of these tumour-associated "antigens" in the membrane of rat sarcomata are summarized below:**

| Antigen | Specificity | Antigenic in syngeneic host | Molecular weight | Antisera used to detect it |
|---|---|---|---|---|
| TSTA | Found only in MC-I and not in embryonic tissue | Yes | 40–50,000 | (a) Serum of syngeneic rats hyperimmunized with MC-I tumour |
| OEA I | Found in many rat sarcomata and in early but not in late embryos | Yes | Unknown | (b) Xenogeneic Antiserum I raised to aqueous extract of MC-I sarcoma and absorbed by normal tissue |
| OEA II | Found in perchloric acid soluble extract of all rat tumours, in embryos and in low concentration in adult tissue | No | 40,000 | Xenogeneic Antiserum II raised to perchloric acid extract of MC-I and absorbed by perchloric acid extracted normal adult tissue |
| OEA III | Found in early embryos, all rat tumours and adult skin | No | 67–70,000 | Xenogeneic Antiserum III raised to aqueous extract of 9–11 day embryos and absorbed by extracts from normal adult tissue |

* Present address: McGill University Medical Clinic, Montreal General Hospital, Montreal, Quebec, Canada.

CHEMICALLY-INDUCED sarcomata in experimental animals have been shown by transplantation tests to have in their membranes a tumour specific transplantation antigen (TSTA) which evokes an immunological host reaction such that following suitable immunization procedures tumour cells are rejected. TSTAs, when assayed by graft rejection and *in vitro* cytotoxicity tests, appear not to cross-react and to be unique for each particular chemically-induced tumour. In addition to these antigens which are unique to each individual tumour there are tumour-associated macromolecules which are common to many tumours; frequently these are substances normally found only in embryonic life. Against virally induced sarcomata of the hamster such embryonic antigens induce graft rejection (Coggin, Ambrose and Anderson, 1971) but in other tumour systems the presence of embryonic antigens has usually been identified by xenogeneic antisera and referred to as tumour-antigens, but this is in some respects a misnomer since they may not be antigenic in the syngeneic host. Stonehill and Bendich (1970) using xenogeneic antiserum, provided evidence for such one " antigen " present in a wide variety of mouse tumours, embryos and adult skin but no other adult tissue. Baldwin, Glaves and Pimm (1971) found that the serum from syngeneic multiparous rats bound to the membranes of many rat tumours, and interpreted this as indicating the presence of an embryonic substance, but this did not appear to be antigenic in the tumour bearing host.

However, hypersensitivity tests showed that chemically induced rat sarcomata contain in addition to the TSTA unique to each tumour, a tumour-specific material common to all sarcomata tested, against which the tumour bearing host reacts (Wang, 1968). This common tumour antigen was shown to be of embryonic origin (Alexander, 1971).

Recently Taranger *et al.* (1972) reported that papillomata and carcinomata induced in the bladder of mice and rats contain a common antigen against which the tumour bearing host mounts a cell-mediated immune response. This antigen appeared to be confined to bladder tumours and was not found in chemically-induced sarcomata and may therefore differ from that reported in sarcomata by Wang (1968) and Alexander (1971).

In the present investigation we found that sera from rats resistant to a syngeneic sarcoma contained, in addition to antibodies to the TSTA, an antibody against a membrane component present in unrelated rat tumours. In other words, there appears to be in the membrane of cells from several different tumours a common component immunogenic to the syngeneic host. This material is also found in rat embryos. The onco-embryonic antigen (OEA) detected by syngeneic immune serum was not the only embryonic material found in the rat sarcoma, since xenogeneic antisera showed in addition to this OEA, two other embryonic materials which do not appear to be immunogenic in the syngeneic host. One was similar or identical to that described by Stonehill and Bendich (1970) and the other has some similarities to the carcino-embryonic antigen (CEA) of human gastro-intestinal tract tumours (Gold and Freedman, 1965). These findings gave us the opportunity to assess the potential role of embryonic antigens and to elucidate the relationship of embryonic " antigens " reported by others using xenogeneic antisera, to that detected by the syngeneic tumour immune serum. This study was also designed to obtain information about the location and some physicochemical properties of the embryonic antigens and to compare these with the TSTA.

### MATERIALS AND METHODS

*Rats.*—Inbred male hooded rats were used throughout and their genetic identity established by skin grafting.

*Tumours.*—A transplanted sarcoma MC-I, originally induced by [20]methylcholanthrene subcutaneously, was selected for study because of its strong immunogenicity and non-cross reaction as judged by standard transplantation tests. All tumours were grown intramuscularly in a hind limb and surgically excised when 2–3 cm in diameter. Early generations were stored at liquid nitrogen temperature and withdrawn at intervals for passage in syngeneic hooded rats and tests were carried out on tumours from generation 5–17. Other tumours used for comparison were spontaneous or were induced by chemicals or irradiation.

*Embryonic tissues.*—These were obtained free of maternal tissue from syngeneic hooded rats at two different gestational periods; 9–11 days and 15–17 days.

*Preparation of tumour, embryo and normal tissue extracts*

(*a*) *Aqueous extraction.*—Tumours freed of surrounding normal and necrotic tissue were stored at −20°C until processed. For the preparation of antigen, tissues were thawed, finely minced with scissors, washed in phosphate-buffered saline, suspended in 3 volumes of distilled water and homogenized for 3–5 min. After centrifugation at 12,000 *g* for 30 min, the supernatant was dialysed against distilled water for 24 hours, clarified by a further centrifugation (at 10,000 *g* for 20 min) and lyophilized. All operations were performed at 4°C. Embryonic, foetal and normal tissues were similarly stored and extracted.

(*b*) *Perchloric acid extraction.*—1·0 mol/l perchloric acid extraction, a technique employed by Gold and Freedman (1965) for isolating CEA of human colon carcinomata, was used to prepare a tumour cell surface glycoprotein extract.

(*c*) *Crude membrane fractions.*—Finely minced MC-I tumour was suspended in a 0·25 mol/l sucrose buffer (2 mmol/l $MgCl_2$, 2 mmol/l, $CaCl_2$, 1 mmol/l $NaHCO_3$) pH 7·6, and homogenized with an ultra-turrax, in a controlled manner so that the nuclei remained intact (Baldwin and Glaves, 1972). The resulting tumour homogenate was centrifuged (at 600 *g* for 12 min) to remove nuclei and debris, and the supernatant was kept. The pellet was resuspended in sucrose buffer and centrifuged, and this step was repeated three times. The pooled supernatants were then centrifuged (at 90,000 *g* for 2 hours) and the pelleted membranes were dispersed in either phosphate buffered saline or 5 mmol/l Tris-HCl buffer, pH 7·6.

(*d*) *Solubilization of membrane antigens.*—(1) To crude membrane fractions obtained by the above method 10 ml of 0·4 mol/l L-cysteine and papain (Sigma Chemical Co. Ltd.) at a concentration of 1 mg/30 mg membrane protein were added. Incubation was for 1 hour at 37°C and the reaction was stopped by iodoacetic acid and NaCl was added to bring the solution to 0·15 mol/l. Following removal of cellular debris by centrifugation (1·5 hours at 90,000 *g*) the supernatant was concentrated with aquacide 1. An alternative method for antigen preparation was also used in which the minced tumour tissues were suspended in 3·5 mol/l KCl (Reisfeld, Pellegrino & Kahan, 1971) and homogenized with an ultra-turrax. The homogenate was gently agitated for 18 hours at 4°C, then centrifuged (2 hours at 90,000 *g*). The solubilized membrane antigens in the supernatant were dialysed against phosphate buffered saline for 24 hours and the DNA precipitate was removed by centrifugation (30 min at 1,500 *g*). The supernatant was concentrated with aquacide 1 as before.

*Preparation of antisera*

(*a*) *Syngeneic antiserum* to the MC-I fibrosarcoma was raised by injecting viable MC-I cells intramuscularly into hooded rats and surgically excising the resulting tumour. The rats then received 6 injections over 3 months of a mechanically prepared and (15,000 rad) irradiated MC-I tumour cell suspension at multiple sites, including intraperitoneal. Rats were bled after the sixth injection and subsequent bleedings were preceded by an additional immunization.

(*b*) *Xenogeneic antiserum.*—The regimen consisted of 3 injections over 2 weeks (at multiple intramuscular sites) of a given lyophilized antigen resuspended in sterile distilled water at 5 mg/ml and emulsified with equal volumes of complete Freund's adjuvant. Booster injections of 2 ml were prepared with incomplete Freund's adjuvant and administered subcutaneously at the fourth week. Blood was collected 2 weeks later and subsequent bleedings were preceded by an additional booster of an emulsion of incomplete Freund's adjuvant and the antigen extract.

*Absorption procedures*

The initial absorption of the xenogeneic antisera was performed with normal rat serum that had been insolubilized by cross-linking with glutaraldehyde (Avrameas and Ternynck, 1969). Further absorptions were by addition of an excess of a lyophilized pool of aqueous extracted syngeneic rat tissue, comprising heart, lung, liver, spleen, gut, skin, skeletal muscle and connective tissue. Approximately 75–150 mg of lyophilized normal tissue extracts were needed to absorb fully 1 ml of xenogeneic antisera. The reaction mixture was incubated for 1 hour at RT, then 18 hours at 4°C when it was clarified by centrifugation (20 min at 20,000 *g*; 4°C). In the instance of xenogeneic antiserum to the MC-I perchloric acid tumour extract, absorptions were performed with perchloric acid extracted normal serum (30–50 mg) and tissues (20–30 mg).

*Assay techniques*

(1) *Indirect membrane immunofluorescence* was performed on viable single tumour cell suspensions obtained from finely minced solid tumour with 0·04% trypsin and 0·04% collagenase in the presence of a small amount of DNAase. When xenogeneic antiserum was tested, lymphocyte suspensions prepared from teased normal hooded rat spleens were used as controls. Xenogeneic antiserum was used at dilutions 1/5 to 1/40. Syngeneic tumour immune serum was tested at dilutions 1/4 to 1/12. The appropriate fluorescein-conjugated antiserum to rat $\gamma$-globulins or rabbit $\gamma$-globulins (Wellcome Reagents) was used at 1 : 12 dilution. In order to quantitate the results, fluorescent indices (FI) were calculated:

$$FI = \frac{\text{\% staining of cells with specific antiserum}}{\text{\% staining of cells with normal serum}}$$

A reaction was defined as positive when the FI was equal to, or greater than 2·5 for both syngeneic and xenogeneic antisera.

(2) *Inhibition of membrane immunofluorescence.*—The antigenic activity in the various samples of lyophilized tissue extracts or their concentrated chromatographic fractions was detected and quantitated by their capacity to inhibit antiserum from binding to its cell surface antigens on viable MC-I tumour cells. The lyophilized antigens were added in equal amounts by weight to the appropriately diluted antiserum. In the instance of antigens already in solution, the antiserum was diluted with this. The mixtures were incubated for 1 hour at RT, then overnight at 4°C and clarified before use (20 min at 4,000 *g*; 4°C). The amount of antibody not complexed to antigen was assayed by membrane immunofluorescence. The presence of specific antigen in the sample reduced the fluorescent index compared with that obtained with antisera alone or antisera incubated with normal tissue extracts.

(3) *Immunodiffusion*—This was performed in 1% Agar (Difco) with the addition of 2·5% polyethylene glycol (6,000 MW) in barbitol buffer (pH 8·2) (Harrington, Fenton and Pert, 1971). The wells had an outside diameter of 5 mm, and 3 mm separation, and these were filled and the slides incubated at 37°C for 24 hours in a moist chamber and continued for 7 days at 4°C to observe the development of additional precipitin lines. The technique of ICD of Darcy (1972) was also employed to detect soluble Ag-Ab complexes. At the time of testing, the lyophilized material was resuspended in a minimum volume of distilled water. Protein concentrations of these final tissue extracts as determined by spectrophotometric absorbence at 280 nm wave length (albumin standard) ranged from 50–60 mg/ml.

(4) *Precipitation-inhibition assay.*—This technique was employed to detect antigens present in concentrations too low to give precipitation reactions by immunodiffusion. The lyophilized material to be studied was added to the antiserum and incubated overnight at 4°C. The control unabsorbed, and the absorbed test antisera were added to the outside wells with the antigen standard in the centre well. The presence of antigen in any lyophilized tissue extract used to absorb the test sera resulted in the retardation of the movement of the precipitation reaction towards the centre well.

(5) *Chromatographic fractionation of extracts.*—Column chromatography was carried out in 4 cm$^2$ × 55 cm columns packed with Biogel A0·5 m, A1·5 and P-150, equilibrated and eluted with 0·1 mol/l Tris, 0·2 mol/l NaCl and 1 mmol/l EDTA buffer titrated to pH 8 with HCl at 4°C. The columns were calibrated with proteins of known molecular weight and a graph of the $E_V/E_0$ against log

MW plotted for each column. Samples collected from one or more runs were concentrated in an Amicon ultra-filtration cell using PM-10 membranes and assayed by immunodiffusion or inhibition of membrane immunofluorescence.

### RESULTS

In this investigation tumour-associated macromolecules were studied principally by the binding of antisera to viable cells. The test therefore only provides data on macromolecules present in or on the plasma membrane of tumour cells. It is quite possible that of the four cell surface components to be described some may also be found in the cytoplasm of the cell and there may be further tumour-associated macromolecules which are found only in the cytoplasm.

(1) *Two tumour membrane components antigenic in the syngeneic host; the TSTA, and an onco-embryonic antigen (OEA I).*

The present investigation began with the finding that serum of rats hyperimmunized with syngeneic MC-I sarcoma contained antibodies to two apparently different antigens in the membrane of the sarcoma cells. As Pilch and Riggins (1966) and Baldwin and Barker (1967) had observed, such sera reacted strongly with the membrane of the tumour used for immunization (Table I). Unlike the finding of these authors, however, the syngeneic serum against the MC-I sarcoma also contained antibody which reacted, albeit very weakly, with the membrane of most of the other rat tumours studied (Table I). On testing the MC-I tumour immune serum, diluted 1/5, on MC-I sarcoma cells in suspension, bright and dense staining approaching confluence was observed on 95–100% of the cells and a FI of 13·0 or greater was continually observed. When the MC-I tumour immune serum, diluted 1/5, was tested on unrelated sarcoma cells a FI of 2·5 or greater was consistently found for most sarcomata. At greater serum dilutions, membrane staining was still noted occasionally but at barely significant levels. Similar results were obtained using a quantitative mixed haemadsorption test with $^{51}Cr$-labelled indicator cells (Thomson, Eccles and Alexander—to be published). Even after extensive absorption of the MC-I tumour-immune serum with normal tissue to ensure that autoantibodies to normal cell components which are found in rat sera (Weir and Elson, 1969) were removed, the FI on unrelated tumours was not lowered.

To study the nature of the common-cross-reacting antigen, MC-I tumour-immune serum was incubated with unrelated MC-3 sarcoma cells or various other tissue extracts. It was then tested on MC-I cells and unrelated MC-3 cells to determine whether the antibody to the cross-reacting antigen had been specifically absorbed (Table II). As the data shows, the 9–11 day gestation embryonic extract lowered the FI index, but the 15–18 day embryonic, the normal tissue, and placental extracts did not. Also, absorption

TABLE I.—*Membrane Immunofluorescence with Syngeneic MC-I Tumour Immune Serum*

| | Fluorescence index | |
|---|---|---|
| Tumour cell suspension | Serum diluted 1/5 | Serum diluted 1/11 |
| MC-1 sarcoma | 13·1 | 9·0 |
| MC-3 sarcoma | 4·0 | 2·9 |
| 10 different $^{20}$MC induced primary sarcomata | 3·8–4·5 | 2·5–2·8 |
| Benzpyrene induced sarcoma (HSH) | 2·6 | 1·5 |
| Benzpyrene induced sarcoma (HSN) | 2·3 | 1·3 |
| Benzpyrene induced sarcoma (HSG) | 2·9 | 2·3 |
| Primary benzpyrene induced sarcoma | 2·9 | 2·4 |
| Imferon-induced sarcoma | 2·9 | 1·9 |

TABLE II.—*Syngeneic MC-I Tumour Immune Serum and Inhibition of Membrane Immunofluorescence with Antigen*

| Tissue extract used for absorption | Fluorescence index MC-I cells[1] | MC-3 cells[2] |
|---|---|---|
| None (PBS) | 9·0 | 4·0 |
| Embryonic Ag | 6·2 | 1·5 |
| Placental Ag | 8·3 | 3·5 |
| Foetal Ag | 8·4 | 3·7 |
| Normal tissue | 8·6 | 3·9 |
| Cells from unrelated MC-3 sarcoma (5 × 10⁷/ml) | 7·6 | 1·1 |
| Cells from an unrelated primary sarcoma (5 × 10⁷/ml) | 7·7 | 1·2 |

[1]Antiserum diluted 1 : 10
[2]Antiserum diluted 1 : 5

with unrelated tumour cells, such as MC-3, consistently lowered the FI for the MC-I sarcoma cells but never by more than 30%. On the other hand, absorption of MC-I tumour immune serum with unrelated sarcoma cells such as MC-3, abolished MC-3 membrane staining. We shall refer to this onco-embryonic antigen as OEA I. Absorption with embryonic material failed to abolish the reaction against the TSTA of the MC-I, and our experiments provide no support for the hypothesis that the TSTA is a substance also present in embryos.

Not unexpectedly, the syngeneic MC-I tumour-immune serum did not produce the usual precipitation reaction when run by gel diffusion against an aqueous extract of the MC-I tumour. In an attempt to obtain a precipitating antibody, rabbits were immunized with an aqueous extract of the MC-I tumour followed by absorption with normal tissue. This Xenogeneic Antiserum I was shown by membrane immunofluorescence to react with the TSTA of MC-I and the OEA I on other rat tumours (Tables III and IV). The Xenogeneic Antiserum I on MC-I cells gave a FI of 8·8 and approximately 4·0 on unrelated tumour cells. It did not stain normal spleen cells. As the data show in Table IV, complete absorption of the Xenogeneic Antiserum I was obtained only with MC-I tumour extract. Embryonic extract and unrelated tumour cell extracts produced a 50% inhibition of the Xenogeneic Antiserum I.

On immunodiffusion a precipitation

TABLE III.—*Membrane Immunofluorescence with Xenogenic Antiserum-I to Whole Aqueous Extract* (*Absorbed*).

| Target Cells | Fluorescence index* |
|---|---|
| Spleen cell suspension | 1·5 |
| Tumour cell suspensions from | |
| MC-I sarcoma | 8·8 |
| MC-3 sarcoma | 4·0 |
| Ten different primary [20]MC induced sarcomata | 3·8–4·9 |
| Two different primary benzpyrene induced sarcomata | 3·4–3·7 |
| HSH } Transplanted benzpyrene induced sarcomata | 3·5 |
| HSG } | 4·6 |
| HSN } | 3·0 |

*Serum absorbed with normal rat serum and normal rat tissues and diluted 1/10.

TABLE IV.—*Xenogeneic Antiserum-I to Whole MC-I Aqueous Extract* (*Absorbed*) *and Inhibition of Membrane Immunofluorescence with Cell Extracts*

| Tissue extract used for absorption | Fluorescence index with* MC-1 sarcoma cells |
|---|---|
| None (PBS) | 8·8 |
| Aqueous extract of normal tissues | 8·5 |
| Aqueous extracts of tumours: | |
| MC-I | 1·7 |
| MC-3 | 4·5 |
| Primary [20]MC induced sarcoma | 5·8 |
| HSH sarcoma | 5·0 |
| Mammary adenocarcinoma | 5·2 |
| Aqueous extract of embryonic tissues | 4·4 |

* Serum diluted 1/10.

line was obtained against an aqueous extract of MC-I. This, however, proved not to be due to reaction with the TSTA or OEA I, but was due to $\alpha_2$ macroglobulin, a serum protein absent in normal serum but synthesized by the liver in response to inflammation or tumours. The rabbit $\alpha_2$ macroglobulin antiserum (kindly supplied by Dr D. A. Darcy) which produced a precipitin line of identity with the Xenogeneic Antiserum I gave no membrane staining of mechanically-prepared MC-I tumour cells in suspension.

(2) *A tumour-associated embryonic material soluble in perchloric acid* (*OEA II*)

Xenogeneic Antiserum II was prepared in order to test if a CEA-like material was present in rat sarcomata and whether this material bore any relationship to OEA I, antibodies against which are present in the syngeneic MC-I tumour-immune serum. On immunodiffusion, the absorbed Xenogeneic Antiserum II gave a single precipitin reaction with either aqueous or perchloric acid extracts of MC-I. A reaction of complete identity was obtained with aqueous extracts of early and late embryos and all solid tumours tested, whether syngeneic or allogeneic (Fig. 1a). No precipitin reaction was obtained with aqueous extracts of individual organs or pooled normal tissues. However, if perchloric acid extracts of individual organs such as skin, gut or liver were prepared and highly concentrated, they gave lines of identity with aqueous MC-I tumour extract. The estimated antigen content of normal and tumour tissues differed by approximately 50-fold. We shall refer to this material as OEA II.

OEA II is not related to the TSTA or to OEA I since it did not inhibit the syngeneic MC-I tumour-immune serum (Table V), which of course contains

(*a*) (*b*)

FIG. 1.—Double immunodiffusion precipitin reactions in 1% agar gel with 3% PEG. Absorbed Xenogeneic antiserum II in centre well, antigen extracts in peripheral wells.

(*a*) 1. Perchloric acid MC-I sarcoma;
2. Aqueous MC-I sarcoma;
3 and 4. aqueous 9–11 and 15–17 day embryo, respectively.
5 and 6. Aqueous unrelated MC-3 sarcoma and mammary adenocarcinoma, respectively.

(*b*) 1. as *a* 1.
2. Perchloric acid gut;
3, 4 and 5. perchloric acid tumour-bearing serum; normal serum and pool of normal adult organs, respectively;
6. Biogel P–150 concentrated fractions tubes 23–27.

FIG. 2.—Double immunodiffusion precipitin reactions in 1% agar gel with 3% PEG. Absorbed Xenogeneic antiserum III in centre well, antigen extracts in peripheral wells.

1. 9–11 day embryo;
2. MC-I sarcoma;
3. Renal tumour;
4. MC-3 sarcoma;
5. Normal tissue;
6. 15–17 day embryo.

antibodies to these two antigens. By using Xenogeneic Antiserum II to stain living cells it was clear that OEA II is associated with the membrane of tumour cells. Its association, however, appears to be less intimate than that of the TSTA or OEA I, since after 0·1% trypsinization for 1 hour the living MC-I cells now showed only minimal staining with Xenogeneic Antiserum II, whereas the presence of TSTA and OEA I could still be readily shown with the syngeneic tumour-immune serum.

As already indicated, the tumour-immune serum is not absorbed by OEA II and hence it would appear that this substance is not antigenic to the rat. OEA II has several of the properties of CEA but it is a much smaller molecule than CEA.

(3) *A further embryonic component (OEA III) on the membrane of sarcoma cells*

Stonehill and Bendich (1970) have demonstrated that antisera raised against aqueous extracts of embryonic tissues cross-reacted with extracts from mouse tumours. Adopting a similar procedure we obtained Xenogeneic Antiserum III raised against aqueous extracts of 9–11 day old embryos and absorbed it with normal adult tissues (excluding skin). This produced a precipitin reaction with a component which was present in aqueous extracts of every rat tumour tested, and gave a line of complete identity with embryonic tissue extract (Fig. 2). This material which will be referred to as OEA III, did not cross-react with OEA II, the antigen extracted from tumours with perchloric acid. In fact, OEA III was not perchloric acid soluble, so that perchloric acid extracted embryos or tumours gave no precipitin reaction when tested with Xenogeneic Antiserum III. Xenogeneic Antiserum III did not give any precipitin lines when run against extracts from normal tissues or 14–18 day embryos. Using inhibition of immuno-precipitation tests, OEA III was detected in low concentrations in aqueous extracts of adult skin and whole 14–18 day embryos.

OEA III is not related to OEA I

TABLE V.—*Inhibition of Membrane Immunofluorescence with Onco-embryonic Associated Components*

| Substances used for absorption | Syngeneic MC-I[1] tumour immune serum MC-I cells | Syngeneic MC-I[1] tumour immune serum MC-3 cells | Xenogeneic antiserum-I[2] MC-I cells |
|---|---|---|---|
| None (PBS) | 9·0 | 4·0 | 8·8 |
| OEA II | 9·1 | 3·9 | 8·1 |
| OEA III partially purified | 9·1 | 3·9 | 8·0 |
| Aqueous extract of whole 9–11 day embryos | 6·2 | 1·5 | 4·5 |

[1]Diluted 1 : 10 for MC-I cells

Diluted 1 : 5 for MC-3 cells

[2]Xenogeneic antiserum-I to aqueous whole extract diluted 1 : 10

because it did not inhibit the syngeneic tumour-immune serum in membrane immunofluorescence (Table V). This finding suggests that OEA III, like OEA II, is not immunogenic in the rat. Xenogeneic Antiserum III, like Xenogeneic Antiserum II, stained the surface of viable tumour cells and we therefore conclude that OEA III is at or near the surface of the tumour cell, but unlike OEA II, it is less readily detached by trypsinization.

(4) *Comparison of the physicochemical properties of the four tumour-associated antigens studied*

(a) *Presence in membrane fractions.*—The relatively crude membrane preparations of MC-I tumour when added to the Xenogeneic Antiserum I and syngeneic MC-I tumour-immune serum completely abolished their capacity to stain viable tumour cells. Similarly, addition of the membrane preparation to Xenogeneic Antisera II and III abolished their capacity to give precipitin lines against the perchloric acid extracts from tumours and the aqueous extract from embryo respectively. Also after absorption with the membrane fractions both Xenogeneic Sera II and III failed to stain living tumour cell membranes. These studies confirm the immunofluorescent findings that TSTA, OEA I, II and III are all associated with the membrane.

(b) *Fractionation of aqueous extracts from MC-I by gel chromatography.*—The aqueous MC-I tumour extract was fractionated on a Biogel column A0·5 m, A1·5 m and P-150, and the different fractions were assayed for antigen content by their capacity to inhibit staining of MC-I tumour cells, or by immunoprecipitation reaction. Xenogeneic Antiserum II gave a precipitin line only with a fraction corresponding to a mol. wt. of 40,000 on Biogel P-150 and it represented the perchloric acid soluble OEA II. Xenogeneic Antiserum III to the aqueous embryonic extract gave an immunoprecipitation reaction with a substance appearing in the mol. wt. range of 65–70,000 and is OEA III.

The syngeneic MC-I tumour-immune serum was absorbed by material appearing in the excluded volume on Biogel A0·5 m and A0·15 m (*i.e.*, of molecular weight greater than $1 \times 10^6$). This high molecular weight material with TSTA and OEA I activity probably consisted of membrane fragments and the data are consistent with the interpretation that OEA II and III are relatively easily detached from the membrane while TSTA and OEA I require special treatments to be released.

(c) *Solubilization of antigens.*—Normal transplantation antigens are released in a soluble form from cells by digestion with papain (Mann *et al.*, 1969) or 3·0 mol/l KCl (Reisfeld *et al.*, 1971). These treatments also release the tumour-associated membrane antigens (Baldwin and Glaves, 1972; Meltzer *et al.*, 1971) and were applied to the MC-I tumour. Both papain and KCl extracts prepared as described contained TSTA, OEA II and III activity as assayed by the capacity to prevent membrane immunofluorescence or give immunoprecipitation with the relevant antisera. When a papain or KCl extract was fractionated on Biogel A0·5 m and P-150, OEA II and III were found in similar fractions as obtained with aqueous extract of MC-I. After papain digestion or KCl extraction, the anti-TSTA activity appeared principally in fractions with a molecular weight of approximately 40–50,000 (Fig. 3). This suggests that papain or KCl released the TSTA moiety from the large molecular weight material of the aqueous tumour extract.

No OEA I activity was found in the papain or KCl extract by inhibition of membrane immunofluorescence, but it is possible that its presence was undetected for quantitative reasons.

The partially purified water-soluble TSTA isolated by exclusion chromatography as described above was immunogenic in the rabbit and produced

Fig. 3.—Biogel A0·5 m (upper) and P–150 (lower) chromatography of the soluble TSTA and OEA active material obtained from papain digests of membranes from MC-I sarcoma. Protein pattern was determined by measuring $A_{280nm}$ (vertical axis, left). Material eluting in volumes prior to tube 12 and 13 was excluded from the Biogel A0·5 m and P–150 respectively. The TSTA activity was determined by testing the contents of pooled tubes in the fluorescence inhibition assay using MC-I tumour-immune serum. OEA activity was determined by precipitin reaction with appropriate xenogeneic antisera.

xenogeneic antibody to the TSTA of the MC-I sarcoma. This antiserum was rendered monospecific to TSTA by selective absorption with cells from the unrelated MC-3 sarcoma.

### DISCUSSION

Neoplastic transformation by chemical carcinogens is accompanied by a series of modifications in the cell surface structure and in this investigation we have characterized in detail some antigenic moieties that appear on the cancer cell surface. It appears that chemically-induced sarcomata express on their cell surface three classes of components which can be distinguished by immunological means: tumour-specific rejection antigens (TSTA); onco-embryonic antigen(s); and other onco-embryonic components not immunogenic in the syngeneic host.

In general, the TSTA of each chemically-induced tumour is unique whether detected by rejection studies or more extensively by *in vitro* examination of tumour immune reactions. In the present study no evidence was found to suggest that the TSTA of MC-I sarcoma is the product of repressed genes.

The TSTA of chemically-induced hepatomata have been solubilized by others (Baldwin and Glaves, 1972; Meltzer *et al.*, 1971), using techniques developed for obtaining water-soluble transplantation antigens. Here, from the MC-I sarcoma, water-soluble TSTA was obtained by the two methods described. In both methods, a similar crude antigenic moiety was found with a mol. wt., estimated by Biogel filtration, of 40–50,000. The soluble TSTA is biologically active as shown by its inhibition of MC-I tumour-immune serum in membrane immunofluorescence and its capacity to raise xenogeneic antiserum. In other experiments (Thomson, Steele and Alexander, 1973) excess soluble TSTA and TSTA-antibody complexes have been detected in the circulation of tumour-bearing animals. In man, Thomson *et al.* (1969) have shown by a radioimmunoassay that colonic tumours also release a cell surface component, CEA, into the circulation. With the ability to obtain water-soluble TSTA it should be possible in the future to define chemically the nature of the tumour-specific rejection antigens.

The second class of components which were found on the tumour cell surface by membrane immunofluorescence were common to most of the tumours studied. The syngeneic antibody to these antigens was specifically absorbed by early embryonic tissues whereas late embryonic and adult tissues did not lower the antibody titres. The OEA I (onco-embryonic antigen) was located on crude membrane fragments but attempts so far to isolate it in a soluble form have been unsuccessful. In a somewhat analogous situation, a few humans with cancer have a precipitating antibody in their sera that defines a new antigen associated with human neoplasia (Edynack *et al.*, 1972). This soluble antigen, named $\gamma$-feto-protein, has been demonstrated to be present in extracts of a proportion of tumours of all histological types and it occurs in serum and some tissues of the foetus. Like the OEA I antigen of the rat the demonstration in man of the $\gamma$-feto-protein is dependent entirely on the use of antibody occurring naturally rather than xenogeneic antiserum. Although the data rule out the possibility of cell surface auto-antigens accounting for the cross-reaction, it may still be possible that the OEA I is represented in normal adult tissues in a cryptic form unavailable to the host immune mechanism.

In man the immune reaction of the host directed against surface antigens (TSTA) of the autochthonous tumour, as assayed by the cytotoxic action of peripheral lymphocyte tumour cells *in vitro*, appears to be directed principally against antigens shared by all the tumours of the same histological type (Hellström *et al.*, 1971). Tumours induced in experimental animals on the other hand both by *in vitro* cytotoxicity tests and by *in vivo* transplantation immunity evoke a reaction directed against a TSTA which is unique for each tumour and there appears to be no cross-reactivity—the only exception reported so far is for bladder papillomata (Taranger *et al.*, 1972). The present investigation may resolve this apparent discrepancy because in the serum of syngeneic rats, antibodies were found which were specific to the MC-I tumour (*i.e.* the MC-I TSTA) and to another surface component (OEA I) which is present in the membrane of all rat sarcomata tested, and in 9–11 day old embryos but not in older embryos or in the placenta. While OEA I does not appear to evoke a degree of resistance sufficient to be detectable by rejection of tumour cells it clearly evokes an antibody response and may also be responsible for cross-reacting delayed hypersensitivity reactions previously reported for rat sarcomata (Wang, 1968; Alexander, 1971). It seems quite possible that when the TSTA is very strong it obscures the immune reaction against antigens of the OEA I type whereas in other situations, and this may conceivably apply to man, unique TSTA may be very weak or absent and measurements of host response therefore focus on the reaction against a cross-reacting antigen like OEA I.

The recurrence of the onco-embryonic antigens in tumours can be ascribed to alterations in the pattern of gene regulation accompanying neoplastic transformation. Similarly, the third class of component expressed on the tumour surface of rat sarcomata may be the result of genetic derepression. In this instance, the onco-embryonic components (OEA II and III) are not antigenic to the host. This probably results from their continuing presence in adult tissues since the genes governing their synthesis are not fully repressed. The universal appearance of these primitive components on the cell membrane may indicate that they have an important role in determining the social behaviour of the tumour cell. At present, however, the biological function of the primitive components is entirely unknown.

We acknowledge with gratitude the advice of Dr D. A. Darcy, and also the expert technical assistance of Miss V. Sellens, Mrs K. Steele, Mrs S. Eccles, Mr P. Avis and Mr A. Gough. This research was supported by grants from the Medical Research Council and the Cancer Research Campaign.

Dr D. M. P. Thomson was in receipt of a Canadian M.R.C. Fellowship.

### REFERENCES

ALEXANDER, P. (1971) A Cross-reacting Fetal Antigen in Primary Chemically-induced Sarcomas of Rats and its Relation to Immunotherapy. In *Proceedings of Conference on Embryonal and Fetal Antigens in Cancer*. Ed. N. G. Anderson and J. H. Coggin. U.S. Atomic Energy Commission CONF-710527. p. 219.

AVRAMEAS, S. & TERNYNCK, T. (1969) The Cross-linking of Protein with Glutaraldehyde, and its use for the Preparation of Immuno-absorbents. *Immunochemistry*, **6**, 53.

BALDWIN, R. W., GLAVES, D. & PIMM, M. (1971) Tumor-associated Antigens as Expressions of Chemically Induced Neoplasia and their Involvement in Tumor-host Interactions. In *Progress in Immunology*. Ed. Amos. New York: Academic Press. p. 907.

BALDWIN, R. W. & BARKER, C. R. (1967) Demonstration of Tumour-Specific Humoral Antibody against Amino Azo Dye Induced Rat Hepatomas. *Br. J. Cancer*, **21**, 793.

BALDWIN, R. W. & GLAVES, D. (1972) Solubilization of Tumour-specific Antigen from Plasma Membrane of an Amino Azo Dye Induced Rat Hepatoma. *Clin. exp. Immunol.*, **11**, 51.

COGGIN, J. H., AMBROSE, K. R. & ANDERSON, N.G. (1971) Immunization Against Tumors with Fetal Antigens. *Proceedings Conference on Embryonal and Fetal Antigens in Cancer*. Ed. N. G. Anderson and J. H. Coggin. U.S. Atomic Energy Commission CONF-710527. p. 185.

DARCY, D. A. (1972) A General Method of Increasing the Sensitivity of Immunodiffusion: its Application to C.E.A. *Clin. chim. Acta*, **38**, 329.

EDYNACK, E. M., OLD, L. J., VRANA, M. & LARDIS, M. P. (1972) A Fetal Antigen Associated with Human Neoplasia. *New Engl. J. Med.*, **286**, 1178.

GOLD, P. & FREEDMAN, S. O. (1965) Specific Carcinoembryonic Antigens of the Human Digestive System. *J. exp. Med.*, **122**, 467.

HARRINGTON, J. C., FENTON, J. W. & PERT, J. H. (1971) Polymer-Induced Precipitation of Antigen-Antibody Complexes: Precipiplex. Reactions. *Immunochemistry*, **8**, 413.

HELLSTRÖM, I., HELLSTRÖM, K. E., SJÖGREN, H. D. & WARNER, G. A. (1971) Demonstration of Cell-mediated Immunity to Human Neoplasms of Various Histological Types. *Int. J. Cancer*, **7**, 1.

MANN, D. L., ROGENTINE, G., FOHEY, J. L. & NOTHENSON, S. (1969) Human Lymphocyte Membrane (HL A) Alloantigens: Isolation, Purification and Properties. *J. Immun.*, **103**, 282.

MELTZER, M. S., LEONARD, E. J., RAPP, H. J. & BORSOS, T. (1971) Tumour Specific Antigen Solubilization by Hypertonic Potassium Chloride. *J. natn. Cancer. Inst.*, **47**, 703.

PILCH, Y. H. & RIGGINS, R. S. (1966) Antibodies to Spontaneous and Methylcholanthrene-induced Tumours in Inbred Mice. *Cancer Res.*, **26**, 871.

REISFELD, R. A., PELLEGRINO, M. A. & KAHAN, B. D. (1971) Salt Extraction of Soluble HL. A Antigens. *Science, N.Y.*, **172**, 1134.

STONEHILL, E. H. & BENDICH, A. (1970) Retrogenetic Expression: The Reappearance of Embryonal Antigens in Cancer. *Nature, Lond.*, **228**, 370.

TARANGER, L. A., CHAPMAN, W. H., HELLSTRÖM, I. & HELLSTRÖM, K. E. (1972) Immunological Studies on Urinary Bladder Tumours of Rats and Mice. *Science, N.Y.*, **176**, 1337.

THOMSON, D. M. P., KRUPEY, J., FREEDMAN, S. O. & GOLD, P. (1969) The Radioimmunoassay of Circulating Carcinoembryonic Antigen of the Human Digestive System. *Proc. natn. Acad. Sci. U.S.A.*, **64**, 161.

THOMSON, D. M. P., STEELE, K. & ALEXANDER, P. (1973) The Presence of Tumour-specific Membrane Antigen in the Serum of Rats with Chemically-induced Sarcomata. *Br. J. Cancer*, **27**, 27.

WANG, M. (1968) Delayed Hypersensitivity to Extracts from Primary Sarcomata in The Autochthonous Host. *Int. J. Cancer.*, **3**, 483.

WEIR, D. M. & ELSON, C. J. (1969) Antitissue Antibodies and Immunological Tolerance to Self. *Arthritis Rheum.*, **12**, 254.